# Limited Durability of Viral Control following Treated Acute HIV Infection

**DOI:** 10.1371/journal.pmed.0010036

**Published:** 2004-10-26

**Authors:** Daniel E Kaufmann, Mathias Lichterfeld, Marcus Altfeld, Marylyn M Addo, Mary N Johnston, Paul K Lee, Bradford S Wagner, Elizabeth T Kalife, Daryld Strick, Eric S Rosenberg, Bruce D Walker

**Affiliations:** **1**Partners AIDS Research Center, Infectious Disease UnitMassachusetts General Hospital and Division of AIDS, Harvard Medical School, Boston, MassachusettsUnited States of America; **2**Howard Hughes Medical Institute, Massachusetts General Hospital and Division of AIDSHarvard Medical School, Boston, MassachusettsUnited States of America; University of OxfordUnited Kingdom

## Abstract

**Background:**

Early treatment of acute HIV infection with highly active antiretroviral therapy, followed by supervised treatment interruption (STI), has been associated with at least transient control of viremia. However, the durability of such control remains unclear. Here we present longitudinal follow-up of a single-arm, open-label study assessing the impact of STI in the setting of acute HIV-1 infection.

**Methods and Findings:**

Fourteen patients were treated during acute HIV-1 infection and subsequently subjected to an STI protocol that required retreatment if viral load exceeded 50,000 RNA copies/ml plasma or remained above 5,000 copies/ml for more than three consecutive weeks. Eleven of 14 (79%) patients were able to achieve viral loads of less than 5,000 RNA copies/ml for at least 90 d following one, two, or three interruptions of treatment. However, a gradual increase in viremia and decline in CD4+ T cell counts was observed in most individuals. By an intention-to-treat analysis, eight (57%), six (43%), and three (21%) of 14 patients achieved a maximal period of control of 180, 360, and 720 d, respectively, despite augmentation of HIV-specific CD4+ and CD8+ T cell responses. The magnitude of HIV-1-specific cellular immune responses before treatment interruption did not predict duration of viremia control. The small sample size and lack of concurrent untreated controls preclude assessment of possible clinical benefit despite failure to control viremia by study criteria.

**Conclusions:**

These data indicate that despite initial control of viremia, durable viral control to less than 5,000 RNA copies/ml plasma in patients following treated acute HIV-1 infection occurs infrequently. Determination of whether early treatment leads to overall clinical benefit will require a larger and randomized clinical trial. These data may be relevant to current efforts to develop an HIV-1 vaccine designed to retard disease progression rather than prevent infection since they indicate that durable maintenance of low-level viremia may be difficult to achieve.

## Abstract 2

Long-term follow-up of 14 patients on HAART during acute infection followed by supervised treatment interruptions shows that most failed to achieve lasting control of viremia.

## Introduction

The use of highly active antiretroviral therapy (HAART) can dramatically prolong the life of individuals infected by human immunodeficiency virus 1 (HIV-1) [1], but early hopes for virus eradication have not been realized [2]. The successful use of HAART is limited by drug-related toxicities, high costs, and drug resistance [3], factors which have led to the development of alternative therapeutic strategies, including the use of supervised, or structured, treatment interruption (STI). This approach, involving recurrent limited exposure to autologous virus, has not been successful in chronic infection [4,5], but has been shown to lead to at least transient containment of viremia after intervention in the acute phase of infection in humans and animals exposed to AIDS-associated retroviruses [6,7,8,9].

In the present study, we performed a detailed longitudinal assessment of the impact of early treatment followed by STIs in patients treated during acute or early HIV-1 infection. The main hypothesis of the study was that early treatment of acute HIV-1 infection followed by STI would lead to immune boosting and subsequent control of viremia without the need for drugs. The primary endpoint was the time to viral rebound above 50,000 copies/ml once or above 5,000 copies for three determinations separated by a week each. The early results of this trial were previously reported, showing that five of eight patients were able to achieve a plasma viral load of 500 copies/ml or less at a median of 6 mo off therapy [6]. The current study investigates the frequency and durability of control achieved with this intervention, with follow-up to a median of 5.3 y after infection, and with an increase in size of the cohort to 14 patients. Our results indicate that, although the majority of patients treated in the acute phase of infection go on to control HIV-1 to less than 5,000 RNA copies/ml plasma for at least 6 mo off therapy, the ability to contain viremia below this level over the long term is maintained in a minority of patients.

## Methods

### Objective

The hypothesis of the study was that early treatment of acute HIV-1 infection would confer immunologic maturation and subsequent control of HIV-1 without the need for ongoing drug therapy. Alternatively, if a breakthrough of virus replication was observed, this would provide a boost in HIV-1-specific immunity after reinstitution of antiviral therapy. The primary endpoint was the time to viral rebound to more than 50,000 copies/ml or viral loads above 5,000 copies/ml for three determinations separated by a week each. The secondary objective was to correlate immunologic and virologic parameters with any observed effects including evolution of HIV-1-specific T helper and cytotoxic T lymphocyte responses. The original study protocol, including the patient consent form and the institutional review board approval, can be found in Protocols S1–S4.

### Study Population

Fourteen patients presenting with acute or early HIV-1 infection were enrolled in this study between July 1997 and January 2000 (Table 1). Acute HIV-1 infection was defined by the presence of HIV-1 RNA in the plasma, a negative or weakly positive HIV-1 antibody by HIV-1/2 ELISA, and the detection of no more than three bands in an HIV-1 Western blot; early HIV-1 infection was defined by a positive ELISA and confirmation of early infection by either detuned negative ELISA or previously known negative ELISA. All participants in the study had symptoms compatible with the acute retroviral syndrome and were treated with HAART (one protease inhibitor and two nucleoside reverse transcriptase inhibitors) within a median of 19 d (range, 9–33) from the onset of symptoms. Study participants were recruited from the Massachusetts General Hospital, the Brigham and Women's Hospital, and the Fenway Community Heath Care Center in Boston. All individuals gave written informed consent to participate, and the study was approved by the respective institutional review boards and conducted in accordance with the human experimentation guidelines of the Massachusetts General Hospital.

**Table 1:**
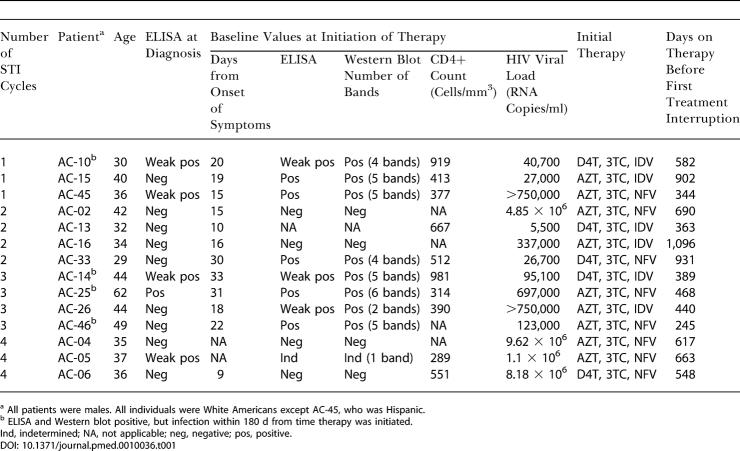
Characteristics of the Study Participants

^a^ All patients were males. All individuals were White Americans except AC-45, who was Hispanic

^b^ ELISA and Western blot positive, but infection within 180 d from time therapy was initiated

Ind, indetermined; NA, not applicable; neg, negative; pos, positive

Six of the 14 individuals were investigated in an interim study [10]. These patients were AC-02 (AS2 in [10]), AC-05 (AS5), AC-14 (AS1), AC-15 (AS3), AC-25 (AS6), and AC-46 (AS4).

### STIs

Entry criteria included treatment with HAART before or shortly after HIV-1 seroconversion, viral suppression on HAART to less than 400 RNA copies/ml for 2 mo, HIV-1 viral load at the time of entry into the study of less than 50 RNA copies/ml, and lack of significant mutations conferring drug resistance [11,12]. Lymphocyte proliferative response to recombinant HIV-1 p24 protein had to exceed a stimulation index of ten before treatment discontinuation, and net counts per minute had to be 800 or greater. All antiretroviral drugs were discontinued simultaneously. After a treatment interruption, patients were seen at least once a week for the first 24 wk, and then monthly, with a total of at least 30 visits for the first year after cessation of therapy. In the second year, visits continued monthly. Treatment was restarted if viral load remained above 5,000 RNA copies/ml plasma for greater than three consecutive weeks, or was in excess of 50,000 copies/ml on any single occasion.

### Human Leukocyte Antigen Typing

High- and intermediate-resolution human leukocyte antigen class I typing was performed at a commercial laboratory (Dynal Biotech, Oxford, United Kingdom) by sequence-specific PCR as described [13].

### Detection of GB Virus C RNA

GB virus C (GBV-C) RNA was detected using a two-step nested PCR amplification reaction from whole plasma RNA. Briefly, GBV-C RNA was extracted from plasma using the Qiagen Viral RNA Mini Kit (Qiagen, Valencia, California, United States) according to the manufacturer's instructions. Extracted RNA was reverse transcribed using the Qiagen OneStep RT-PCR Kit and amplified by nested PCR; in both steps primers specific for the 5′ UTR of GBV-C were used, as described previously [14].

### Chemokine Receptor Genotyping

In order to analyze the chemokine receptor (CCR) 5Δ32 deletion polymorphism, the region spanning the 32-nt deletion was amplified by PCR, and the two alleles were separated by gel electrophoresis [15]. The CCR2–64I polymorphism was detected by PCR–restriction fragment length polymorphism analysis as described previously [16].

### Synthetic HIV-1 Peptides

We synthesized 410 synthetic peptides 15–20 amino acids long at the Massachusetts General Hospital Peptide Core Facility on an automated peptide synthesizer using Fmoc technology, as described [17]. Peptides overlapped by 10 amino acids and spanned the entire HIV-1 clade B 2001 consensus sequence.

### ELISPOT Assays

ELISPOT assays were carried out as described previously [18]. Peripheral blood mononuclear cells (PBMCs) were incubated overnight at 50,000 to 100,000 cells/well in 96-well polyvinylidene plates that had been precoated with 0.5 μg/ml anti-human interferon-γ monoclonal antibody (Mabtech, Stockholm, Sweden). The final concentration of the peptide per well was 14 μg/ml. The numbers of spots per well were counted using an automated ELISPOT plate reader (AID EliSpot reader system, Autoimmune Diagnostika, Strassberg, Germany). A response was considered positive if there were more than 50 spot-forming cells (SFCs)/10^6^ PBMCs and if the well had at least three times the mean number of SFCs in the three control wells. The dependence of responses on CD8+ T cells was determined by measuring the depletion of CD4+ T cells using the Minimacs cell depletion system (Miltenyi Biotec, Bergisch-Gladbach, Germany). When HIV-1-specific CD8+ T cell responses were detected against adjacent peptides, and therefore might represent targeting of the overlap region, responses to the weaker peptide were excluded for calculations of magnitude and breadth, as previously described [19].

### Proliferation Assays

Freshly isolated PBMCs (10^5^ cells) were incubated with baculovirus-derived recombinant p24 protein (Protein Sciences, Meriden, Connecticut, United States) at 5 μg/ml for 6 d and then pulsed with ^3^H thymidine at 1.0 μCi for 6 h before harvesting as previously described [20]. A stimulation index of five or greater was considered significant.

### Statistical Analysis

Time to failure during the different treatment interruptions was assessed by Kaplan-Meier analysis. Patients who were still controlling viremia at the time of last visit, who failed because they restarted therapy without meeting the criteria of virologic failure, or who were lost in follow-up were included in the analyses, but the data were censored at the last evaluable time point. Equality of survival distributions for the first and second treatment discontinuations was evaluated using the Wilcoxon matched-pairs signed-ranks test. CD4+ T cell losses were calculated on regression lines based on least squares fit. A Cox proportional hazards regression model was used for the analysis of continuous variables such as days following onset of symptoms, CD4+ T cell count, HIV viral load, and time to rebound of viremia, as well as for the estimation of hazard ratios for the categorical variables of ELISA, Western Blot, coreceptor polymorphism, and GBV-C status. Statistical analyses of CD8+ and CD4+ T cell responses were based on a Student's *t* test, a multiparametric ANOVA test, a Wilcoxon matched-pairs signed-ranks test, or a Mann-Whitney *U* test, as indicated. *p*-values lower than 0.05 were considered to indicate statistical significance, and all reported *p*-values are two-sided. Statistical analysis and graphical presentation were performed using SPSS, SAS, and Prism software packages.

## Results

### Longitudinal Assessment of Control of Viremia following Treated Acute or Early Infection

**Figure 1:**
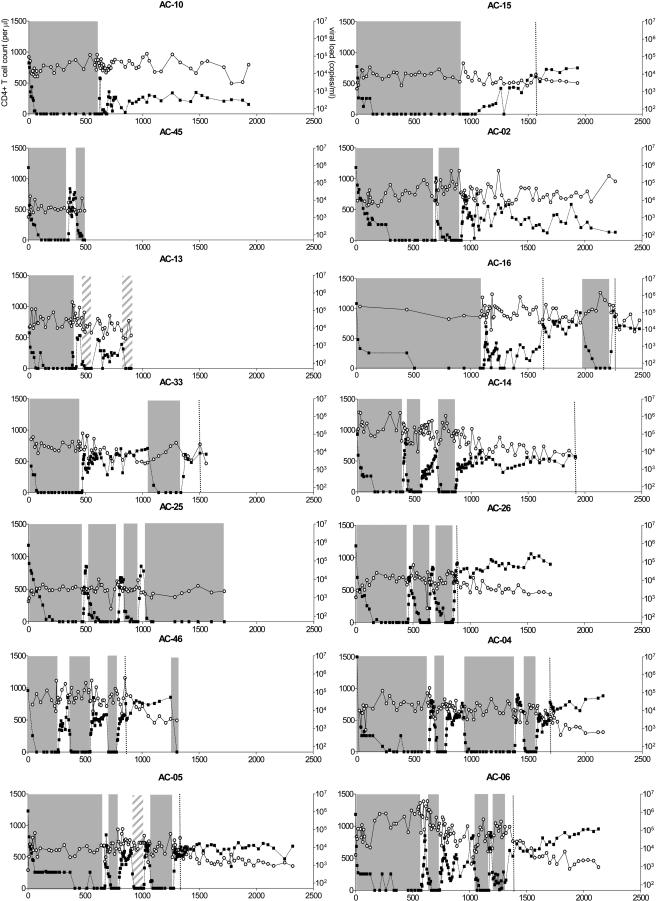
HIV-1 Viral Loads and CD4+ T Cell Counts in the 14 Study Participants Time zero corresponds to first institution of highly active antiretroviral therapy (HAART). Closed squares, HIV-1 plasma viral loads; open circles, CD4+ T cell counts; shaded areas, treatment with HAART according to protocol; diagonally shaded areas, patient restarted therapy without meeting criteria of virological failure; vertical dotted lines, virological failure without reinstitution of HAART. Patients are ordered by increasing number of supervised treatment interruptions.

Fourteen patients identified at the time of acute or early infection (Table 1; Figure 1) were entered into this protocol, and they were followed for a median of 5.3 y from the time of infection (range, 494–2,475 d). Patients underwent successive treatment interruptions after an initial treatment period of at least 8 mo (median, 508 d; range, 245–1,096 d) and were required to restart therapy when viral load exceeded 50,000 RNA copies/ml plasma on a single occasion, or 5,000 copies/ml for three consecutive weeks. For purposes of analysis, patients who dropped out of the study or who reinitiated therapy without meeting criteria were considered to have lost the ability to contain viremia.

Using these criteria for reinitiation of therapy and to define failure, 11 of 14 patients (79%) were able to achieve virologic control to less than 5,000 RNA copies/ml plasma for at least 90 d after one, two, or three treatment interruptions (Table 2). The period of longest containment was after one interruption for five patients, after two interruptions for eight patients, and after three interruptions for one patient (Table 3).

**Table 2:**
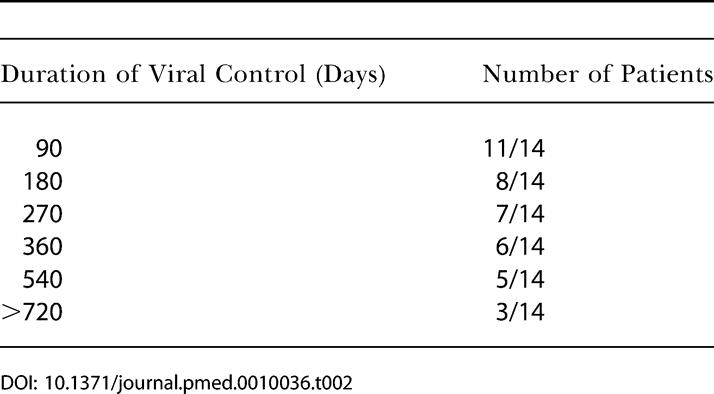
Period of Viral Control Achieved Off Therapy

**Table 3:**
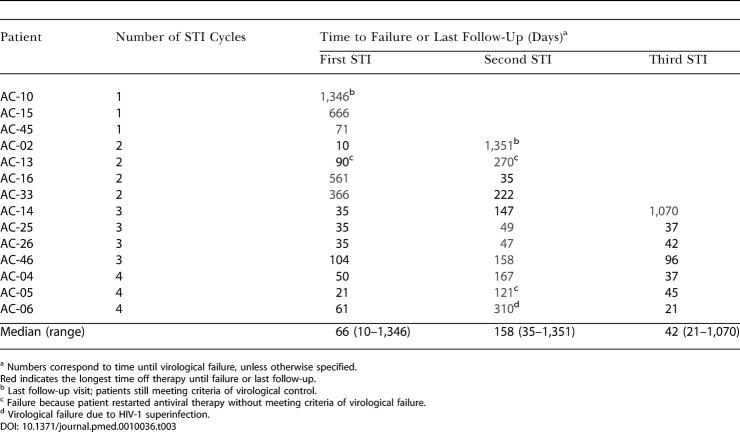
Time to Failure during the STIs

^a^ Numbers correspond to time until virological failure, unless otherwise specified

Red indicates the longest time off therapy until failure or last follow-up

^b^ Last follow-up visit; patients still meeting criteria of virological control

^c^ Failure because patient restarted antiviral therapy without meeting criteria of virological failure

^d^ Virological failure due to HIV-1 superinfection

Once control was achieved, the majority of the patients experienced a subsequent rise in viremia. The median time between cessation of therapy and rebound of viremia (having a viral load greater than 50 copies/ml) was 17 d (range, 7–169 d).

**Figure 2:**
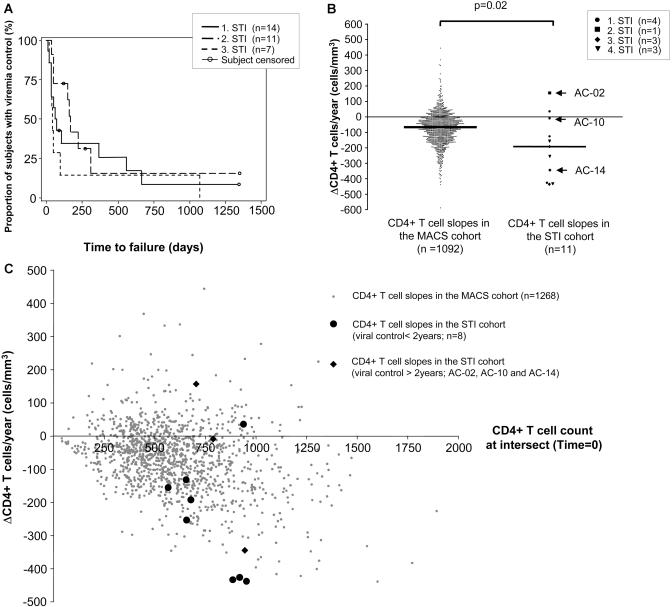
Evolution of Viral Load and CD4+ T Cell Counts during STI (A) Survival curves of time to virologic failure during the first three supervised treatment interruptions. Virologic failure was defined as having a viral load of greater than 5,000 copies RNA/ml plasma for 3 wk or greater than 50,000 copies once. Patients still achieving viral control at the last visit and individuals restarting therapy without meeting criteria or lost in follow-up are censored at the last evaluable time point. The horizontal axis represents the time off therapy since the beginning of the treatment interruption, the vertical axis corresponds to the number of patients maintaining control of viremia. The curves for first, second, and third STIs do not differ significantly from each other (log-rank test, *p >* 0.05). (B) Evolution of CD4+ T cell counts during the longest treatment interruption. Slopes of CD4+ T cell counts during the first year of the longest treatment interruption are shown for patients who experienced a cessation of therapy of at least 12 mo (all except AC13, AC25, and AC45), compared to the natural decline of CD4+ T cell counts in untreated patients of the MACS cohort with early chronic HIV-1 infection (CD4+ counts of >350 cells/mm^3^). CD4+ T cell losses were calculated on a regression line based on least squares fit. The two groups differed significantly from each other (Mann-Whitney *U* test, *p =* 0.02). (C) CD4+ T cell count at intercept and CD4+ T cell slopes during the longest treatment interruption. The CD4+ T cell slopes of the same 11 patients shown in (B) and of untreated patients of the MACS cohort are represented according to the CD4+ T cell count at the intercept of the regression line based on least squares fit with the vertical axis (day 0 of treatment interruption).

Six of 14 patients (43%) achieved a period of control after stopping therapy for 1 y, but only three of 14 (21%) were able to control viremia off therapy at less than 5,000 RNA copies/ml plasma for more than 2 y. Duration of viremia control during successive treatment interruptions was highly variable, and there was no increase in the sustainability of viral containment during successive STI cycles. The three patients achieving control of viremia for more than 2 y did so during the first (AC-10), the second (AC-02), and the third (AC-14) treatment interruption, respectively (Figure 2A). A paired comparison (Wilcoxon matched-pairs signed-ranks test) showed no significant difference in the length of viremia control with subsequent treatment interruptions. Although patients experienced rebound viremia with discontinuation of therapy, none of the patients experienced recurrence of symptoms associated with acute HIV-1 infection.

These data show that at least transient control of viremia to less than 5,000 RNA copies/ml plasma was achieved in the majority of study participants during at least one of the treatment interruptions, but that durable viral control in participants following treated acute infection occurred infrequently. Moreover, the data do not show a consistent pattern of augmentation of viral control with sequential treatment interruptions.

### Effect of Treatment Interruptions on CD4+ T Cell Counts

Although viral load is a strong predictor of disease progression, CD4+ T cell loss is an additional, independent predictor [21]. Early treatment of acute HIV-1 infection led to normalization of CD4+ T cell counts in most patients (median, 753 cells/mm^3^; range, 492–986), but the effect of treatment interruption was variable, even in those doing well, as defined by sustained low viral loads. Overall, 11 of 14 patients interrupted therapy for at least 12 mo, and these individuals were evaluated regarding the effect of treatment interruption on CD4+ T cell loss (Figure 2B and 2C). The rate of change in CD4+ T cell counts during the first year of the longest period off treatment ranged from +157 to −438 cells/mm^3^/y (median, −192). Of the three patients who did not meet viral load criteria for restarting therapy for more than 2 y, one (AC-02) had an increasing CD4+ T cell count of 157 cells/mm^3^/y, one (AC-10) had a stable CD4+ T cell count (−9 cells/mm^3^/y) , and one (AC-14) experienced a decline of 344 cells/mm^3^/y. Comparison with data from the Multicenter AIDS Cohort Study (MACS) showed that the kinetics of CD4+ T cell loss was faster (Mann-Whitney *U* test , *p =* 0.02) than in untreated patients with early chronic HIV-1 infection (average loss of −67 cells/mm^3^/y in patients with a CD4+ T cell count of more than 350 cells/mm^3^ at baseline). However, CD4+ T cell loss rate was in the same range as what has been described after treatment interruption in chronic HIV-1 infection [22,23]. Analysis of CD4+ T cell decline during the second year for the three individuals who controlled viremia for more than 2 y revealed similar trends in CD4+ T cell slopes, although they were less steep: AC-02, +88 cells/mm^3^/y; AC-10, +44 cells/mm^3^/y; and AC-14, −110 cells/mm^3^/y. When the first 3 mo off therapy were excluded in order to minimize the potential effects of recent treatment on CD4+ T cell number, the rate of change in CD4+ T cell counts during the first year off therapy no longer differed statistically from the MACS data (median, −207 cell/mm^3^/y; range, +119 to −699; Mann-Whitney *U* test, *p =* 0.07). A possible reason for steep CD4+ T cell slopes may be high CD4+ T cell counts at time of treatment interruption. Comparison with MACS data (Figure 2C) showed that several of the study participants still behaved as outliers when this factor was considered. These results indicate that periods of relative control of viremia were associated with declining CD4+ T cell counts in most patients.

### Correlation of Clinical and Genetic Markers with Duration of Viremia Control

Although the study was small, we evaluated clinical and laboratory parameters to see if any was predictive of duration of viral control. Analyses included clinical and laboratory parameters at time of presentation with acute HIV-1 infection, genetic markers associated with different rates of disease progression, and the presence or absence of GBV-C coinfection. All patients presented with symptomatic acute infection. Time between onset of symptoms and institution of therapy did not affect duration of control following STI (Cox proportional hazards regression model, *p >* 0.05). The individuals who controlled viremia for a longer time either during the first STI or during any of the treatment interruptions were not different from those who experienced earlier breakthrough as measured by ELISA and Western blot status at initiation of HAART, coreceptor polymorphisms (CCR5delta32, CCR2 V64I), or the presence or absence of GBV-C coinfection (Cox proportional hazards model, *p >* 0.05 in all comparisons; data not shown). The only parameter that was predictive of prolonged viral control during the first treatment interruption was a low viremia at time of institution of therapy (*p =* 0.01): there was a 2.8-fold increase in hazard per order of magnitude increase in viral load. This factor was no longer predictive when the period of longest control of viremia was considered. The time to rebound of viremia (>50 copies/ml or >400 copies/ml) did not correlate with the duration of viral control. Although 11 out of 14 individuals achieved at least transient control of viremia, and three experienced prolonged control, none of these patients possessed the HLA alleles B27 or B57 associated with better disease outcome [24,25].

### Relationship of Magnitude and Breadth of HIV-1-Specific CD8+ T Cells to Duration of Viremia Control

**Figure 3:**
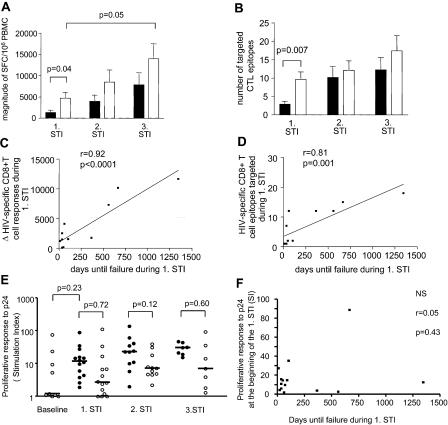
Evolution of HIV-1-Specific CD4+ and CD8+ T Cell Responses during STI (A–D) Magnitude and breadth of increase of HIV-specific CD8+ T cells during supervised treatment interruptions. (A and B) Magnitude (A) and breadth (B) of HIV-specific CD8+ responses at the first day of treatment interruption (black bars) and at the last day off therapy (white bars). Data represent the mean and standard deviation. (C and D) Correlation between the increase of the magnitude (C) or breadth (D) of CD8+ T cell responses and the time off therapy during the first treatment interruption. (E and F) Evolution of CD4+ T helper cell responses during supervised treatment interruptions. (E) Magnitude of CD4 T helper cell responses at baseline and at the first day of treatment interruption (closed circles) and last day off therapy (open circles). Horizontal bars correspond to median values. An stimulation index greater than five was considered significant. (F) Correlation between the magnitude of p24-specific lymphocyte proliferative responses at the beginning of the first treatment interruption and the time patients were able to remain off therapy during the subsequent STI.

To assess the relationship between the clinical outcome and evolution of HIV-1-specific CD8+ T cells, we longitudinally analyzed the breadth and magnitude of CD8+ T cell responses using an interferon-γ ELISPOT and a panel of 410 overlapping peptides spanning the entire HIV-1 clade B consensus sequence. At the beginning of the first STI, HIV-1-specific CD8+ T cells were weak (median of 590 SFCs/10^6^ PBMCs) (Figure 3A) and narrowly directed at a median of two epitopes (Figure 3B). CD8+ T cell responses increased significantly (*p <* 0.05) during the first off-treatment period, reaching a median total magnitude of 2,725 SFCs/10^6^ PBMCs and targeting a median of eight epitopes, and then were sustained when therapy was reintroduced. A further increase in the magnitude and breadth of HIV-1-specific CD8+ T cells was observed in the subsequent off-treatment periods, although these augmentations failed to reach statistical significance. The CD8+ T cell–mediated immune responses emerging during these consecutive cycles of treatment interruption were broadly directed, targeting all structural and most accessory and regulatory HIV-1 gene products (data not shown). However, the magnitude of HIV-1-specific CD8+ T cell responses at the beginning of the first (*r =* 0.01, *p =* 0.76), second (*r =* 0.16, *p =* 0.54), or third (*r =* 0.1, *p =* 0.55) treatment interruptions was not predictive of the time the study participants were subsequently able to stay off therapy according to study criteria.

The periods off treatment allowed for assessment of the relationship between exposure to virus and evolution of immune responses. There was a highly significant positive association between time until virologic failure during the first treatment interruption and change in the magnitude of HIV-1-specific CD8+ T cell responses (*r =* 0.92, *p <* 0.001) (Figure 3C). Similarly, the longer a patient remained off therapy during the second and third interruptions, the greater the augmentation of the total magnitude of HIV-1-specific CD8+ T cell responses (*r =* 0.83, *p <* 0.016; *r =* 0.74, *p =* 0.05, respectively). The increase in CD8+ T cell epitopes targeted during the first treatment interruption was also linearly correlated to the duration until virological failure (*r =* 0.81, *p <* 0.001) (Figure 3D). However, no significant relationship was observed between the augmentation of epitopes targeted during the second and third treatment pauses and the time the study participants were able to remain off therapy in the respective treatment interruption. These data suggest that the duration of a treatment interruption, and therefore the duration of exposure to plasma virus, correlates positively with the magnitude and breadth of HIV-1-specific CD8+ T cell responses that emerge during off-therapy time periods. Yet, CD8+ T cell responses prior to treatment interruptions were not significantly predictive of the duration of time that patients are able to spontaneously control HIV-1 replication, as defined by the study criteria.

### Relationship of Magnitude of Lymphocyte Proliferative Responses to p24 Antigen to Duration of Viremia Control

We next analyzed evolution of lymphoproliferative responses to recombinant HIV-1 p24 Gag protein in order to assess HIV-1-specific CD4+ T cell function. Most individuals had no detectable response at baseline prior to treatment, consistent with prior reports of patients with acute HIV-1 infection [20]. After initiation of therapy, all individuals generated HIV-1-specific lymphoproliferative responses (Figure 3E), which was a criterion for inclusion in the study. During treatment interruptions, there was a variable decline in magnitude, and comparisons between responses on the first day of treatment interruption and last day off therapy did not reach statistical significance (first STI, *p =* 0.72; second, *p =* 0.12; and third, *p =* 0.60, respectively). These HIV-1-specific CD4+ T cell responses also tended to rise with reinitiation of therapy, and some of them were very robust, with stimulation indices over 50 detected in several individuals (Figure 3E). Similar to CD8+ T cell responses, the magnitude of HIV-1-specific CD4+ T helper cell responses at the beginning of the first (*r =* 0.05, *p =* 0.43) (Figure 3F), second (*r =* 0.16, *p =* 0.54), or third (*r =* 0.1, *p =* 0.55) treatment interruption was not statistically predictive of the time the study participants were subsequently able to stay off therapy according to study criteria.

## Discussion

Although early treatment of acute HIV-1 infection followed by treatment interruptions may enhance control of viremia [6,8], the durability of this control remains unclear. Here we analyzed the long-term impact of initiation of antiviral therapy during acute HIV-1 infection followed by STIs in a cohort of 14 patients. Although initial control of viremia to less than 5,000 RNA copies/ml plasma was achieved in the majority of the individuals studied, a gradual increase in viremia and decline in CD4+ T cell counts was observed in most patients, even after a year or more of viral containment. Durable virologic control occurred infrequently, despite the presence of robust HIV-1-specific CD4+ and CD8+ T cell responses detected by standard assays. Moreover, even during periods of successful control of viremia, progressive loss of CD4+ T cells was frequently observed. These data indicate that although early treatment of acute and early infection is frequently associated with transient control of viremia after STI, ongoing low-level viral replication is associated with ultimate virologic breakthrough in most patients.

The standard immunologic assays and virologic assessments in this cohort revealed considerable heterogeneity among the study participants, and did not show a consistent pattern in duration of viremia control during successive treatment interruptions. Eleven of 14 patients (79%) were able to maintain a viral load of less than 5,000 copies/ml for at least 90 d, but progressive loss of control ensued in the majority of patients and only three patients (21%) were able to maintain control for more than 2 y. These three patients did so during the first (AC-10), the second (AC-02), and the third (AC-14) STI. Clinical, genetic, and immunological parameters did not distinguish these three individuals from the other 11 patients, nor did they predict the duration of control following treatment interruption. Indeed, the longer a patient was off therapy, the stronger and more broadly directed the CD8+ T cell responses became, but these were still not sufficient to maintain prolonged control in most patients. Although three patients did not complete the study as initially intended (patient AC-45 withdrew from the study after viral breakthrough on the first STI, AC-13 restarted therapy despite a viral load of less than 5,000 copies/ml during both the first and second STIs and then withdrew, and AC-05 restarted therapy prematurely during the second STI but then failed to control during the third STI), the results are not substantially different if these three are censored rather than considered to have failed to control.

Loss of viral control in this cohort occurred not only in the presence of strong CD8+ T cell responses, but in most cases also in the presence of virus-specific CD4+ T cell responses, although the CD4+ T cell responses often declined during periods of viremia. In addition, total CD4+ T cell numbers were also monitored and declined in most patients over time, including one of the three patients who were able to maintain low viral loads for at least 2 y. Mechanisms leading to rapid CD4+ T cell loss need to be further studied in future STI trials. Other parameters including chemokine receptor polymorphisms [26] and GBV-C coinfection [27,28] similarly failed to explain the different courses following treatment interruption. The only parameter found to be associated with longer control of viremia during the first treatment interruption was a lower viral load at time of institution of antiviral therapy. Given the multiplicity of comparisons made, the true significance of this finding is uncertain.

The reasons for progressive loss of control despite augmentation of virus-specific CD4+ and CD8+ T cell responses remain to be defined. In one individual (AC-06), HIV-1 superinfection in the setting of strong and broadly directed HIV-specific cellular immune responses was associated with the loss of viral control, as previously reported [29]. No other cases of superinfection have been identified in these patients (data not shown). The immunologic studies performed failed to show an association between increases in viral load and loss of immune responses, but this may be due to the use of the current standard IFN-γ assays to quantify immune function. Numerous studies now indicate that IFN-γ production alone is not associated with viral load [19,30,31] but rather that functional characteristics of CD4+ and CD8+ T cells may be better associated with viral control [32,33,34,35]. Such studies will be important to pursue. In particular, even a low level of viremia correlates with a low or undetectable frequency of interleukin-2-producing HIV-1-specific memory CD4+ T cells endowed with proliferative capacity in vitro [36,37,38,39], thus abrogating CD4+ T cell help crucial to maintain efficacy of CD8+ T cell functions. In an interim study of a subset of six of the 14 patients presented here (patients AC-02, AC-05, AC-14, AC-15, AC-25, and AC-46), a fully differentiated effector phenotype of HIV-1-specific CD8+ T cells for selected epitopes was found to be associated with better control of viremia [10]. Other factors that may contribute include functional defects in antigen-specific cell-mediated immunity [35,37,40,41,42], and progressive immune escape [43,44,45]. HIV-1-specific humoral immunity can also affect viral control after treatment interruption [46], and viral factors including viral fitness [47,48] and infection with multiple viral variants [49] can influence viral set point and the rate of disease progression. Virus sequencing studies currently in progress in this cohort indicate that viral breakthrough is associated with sequence changes within and outside known CTL epitopes (data not shown). Full evaluation of the relationship between immune escape and viral breakthrough will require extensive additional analyses, including detailed analysis of responses to autologous virus [50,51]. Assessing the changes in CD4+ and CD8+ T cell functions over time as well as viral evolution under immune selection pressure will be important to evaluate immune correlates in this cohort.

These data are important in light of other recent data on treatment interruption in both acute and chronic infection. In chronic HIV-1 infection, STI studies showed only marginal, if any, improvements of HIV-1 viremia control following a number of treatment interruptions cycles, despite at least transient increases in HIV-1-specific CD8+ and CD4+ T cell responses [4,5,52,53,54,55]. In the setting of infection with a multidrug-resistant virus, this strategy may even be deleterious [56]. Other studies of STI after treated acute HIV-1 infection have shown limited benefits [9], including recent trials such as the PrimSTOP trial [57] and the QUEST study [58]. However, little is known about the relationship between scheduling of HAART and treatment interruptions and the characteristics of viral rebound after therapy has been discontinued.

Although durable control of viremia was not achieved, it is noteworthy that the majority of patients were able to achieve transient relative containment of viremia, providing rationale for future studies aimed at further enhancing immune control. Early treatment alone should still be considered an important therapeutic option. Therapeutic vaccinations administered after treated acute HIV-1 infection and before cessation of therapy have given disappointing results thus far [9], but the availability of new and more potent immunogens requires reassessment of this approach. Indeed, the ability to enhance CD4+ T helper cell responses in the chronic phase of infection has been demonstrated [59], but whether this will enhance CD8+ T cell function requires additional studies. Some promising results have been obtained using immunomodulatory drugs, including cyclosporine [60] and hydroxyurea [61], in combination with antiviral therapy, presumably because of the limitation of T cell activation. Administration of granulocyte-macrophage colony-stimulating factor blunted the viral rebound following interruption of HAART, and largely prevented a decrease of CD4+ T cell counts in an STI trial in chronic HIV-1 infection [62]. These additional therapeutic interventions deserve further investigation in future STI studies.

Although the present study shows progressive viral breakthrough, it was not designed to address whether there might be a change in set point viremia achieved or overall clinical benefit through transient early treatment of acute HIV infection. The definition of failure chosen for this study was a viral load of greater than 5,000 RNA copies/ml plasma, which at the time the study was initiated corresponded to the level of viremia at which treatment was recommended. Larger randomized trials will be needed to determine the potential clinical and virologic benefit of approaches based on STIs. In studies of untreated infection, there is only a 5-fold difference in viremia separating the quartile with the slowest disease progression from the quartile with the most rapid progression [63], suggesting that small differences in steady-state viremia may influence clinical outcome. In the meantime, STI probably should be avoided outside the setting of controlled clinical trials. The data in this study may also be relevant to current efforts to develop a therapeutic AIDS vaccine designed to retard disease progression rather than prevent infection, since they suggest that durable maintenance of low-level viremia may be difficult to achieve.

## Supporting Information

Protocol S1 Study Protocol (68 KB DOC).

Protocol S2 Protocol Amendment (47 KB PDF).

Protocol S3 Patient Consent Form (187 KB PDF).

Protocol S4 Institutional Review Board Approval (36 KB PDF).

**Patient Summary**

**Background**

Highly active antiretroviral therapy (HAART), which is used to treat patients with HIV, can have nasty side effects and is expensive. As a result, for the last five years scientists have been trying to determine if it is possible to give patients breaks from taking antiretroviral drugs, without patients' health suffering. It is likely that such a treatment strategy (called “supervised treatment interruption,” or STI) will not work in patients who have been infected with HIV for a long time. However, until now, the jury was out about whether STI could be of benefit to patients who had only recently been infected with HIV. Some research suggested that if newly infected patients had short “holidays” from taking HAART, it might help to boost their immune system—which in turn might help to keep HIV at bay.

**What Did the Researchers Do and Find?**

The researchers studied 14 patients who had recently been infected with HIV. Patients were treated until their viral load was below the limit of detection by using a very sensitive method. As they were then watched after treatment interruption, most of them were able to maintain a low, though detectable, viral load for some time. The researchers then stopped the treatment and carefully watched the patients over a period of up to five years. If the number of viruses in the patients' blood rose too high, the researchers gave the patients HAART again until the number of viruses fell again. In about half the patients, treatment needed to be restarted within a year because the viruses had started becoming more numerous in the blood. The doctors had hoped that it would take much longer for the number of viruses to reach this level. In other words, giving patients “drug holidays” did not help to keep the virus at bay for long periods of time.

**What Are the Limitations of the Study?**

It is relatively rare for doctors to be able to diagnose HIV shortly after an individual becomes infected with the virus. Many people have HIV for a long time before they see a doctor. Therefore, even if the STI strategy had worked, it would only have been relevant to a few patients. In addition, the study was small and preliminary, so we have to be careful about reading too much into the results. A limitation in the study is that it did not address whether there might be a long-term clinical benefit despite a gradual increase in viral load.

**What Does This Study Mean for Patients?**

It is important to remember that this was an experimental trial—patients with HIV should not stop taking antiretroviral drugs unless their doctor specifically tells them to do so. Although it looks as though STI may not work in the way everyone had hoped, these results may help scientists develop an HIV vaccine that is designed to keep the disease at bay. Whether early treatment of acute infection has an overall benefit in terms of time-to-development of AIDS or need for long-term treatment after drug discontinuation will need to be answered in larger clinical trials designed to answer these important questions.

**Resources on the Web.**

AIDSinfo: http://www.aidsinfo.nih.gov/

AIDSmap: http://www.aidsmap.com/

Medline Plus AIDS Information: http://www.nlm.nih.gov/medlineplus/aids.html

## Abbreviations

CCR: chemokine receptor
GBV-C: GB virus C
HAART: highly active antiretroviral therapy
HIV-1: human immunodeficiency virus 1
MACS: Multicenter AIDS Cohort Study
PBMC: peripheral blood mononuclear cell
SFC: spot-forming cell
STI: supervised treatment interruption
