# Supplementary material for: Limited Durability of Viral Control following Treated Acute HIV Infection
Source: PLoS Med. 2004 Oct 26;1(2):e36. doi: 10.1371/journal.pmed.0010036 (PMC524377; doi:10.1371/journal.pmed.0010036)
Supplement: Protocol S1 — (68 KB DOC). [file pmed.0010036.sd001.doc]

4/5/99

Updated 3/16/00

Updated 4/18/00 FINAL VERSION 7-18-00

4-8-02 ek

4-19-02ek

3-4-03 ek (Closed to enrollment, active follow up continues)

5/28/03

3-2-2004 NO CHANGES

**DETAILED PROTOCOL:**

PRINCIPAL INVESTIGATOR: Eric Rosenberg, M.D.

Co- Principal Investigator: Bruce D. Walker, M.D.

TITLE OF STUDY: Structured therapy interruption in persons with acute HIV-1 infection

FUNDING: AIDS Research Center – NIH

1. BACKGROUND AND SIGNIFANCE:
2. Historical background:

In animal models of chronic viral infection, virus-specific helper cells are essential to maintenance of effective immunity. In the absence of CD4 cells, acute infection leads to transient CTL responses and lowering of viral load, but the CTL response subsequently wanes and virus replication increases (Matloubian, Concepcion et al. 1994; vonHerrath Yokoyama et al. 1996; Azjac, Blattman et al. 1998). In HIV-1 infection, the earliest and most glaring hole identified in the immune repertoire is the absence of virus-specific T helper cell (Th) responses (reviewed in Kalams and Walker, 1998). Rarely have these responses been detected in person with chronic HIV-1 infection, and when they have been detected the levels have been low, with stimulation indices greater than 5 having rarely been reported (Berzofsky, Bensussan et al. 1988; Wahren, orfeldt-Mansson et al. 1989; Schwartz, Sharma et al. 1994; Pontesslli Carlesimo et al. 1995; Beretta, Weiss et al. 1996).

1. Preclinical and clinical studies:

Given the central importance of Th cells in antiviral immunity, we examined the HIV-1-specific T helper cell response in person infected with HIV for up to 19 years who have maintained stable CD4 cell counts and viral loads at the limits of detection by the most sensitive quantitative assays available, and compared these to persons with chronic progressive infection. In non-progressing persons, no or very weak virus-specific Th cell responses were detected, consistent with what has previously been reported in persons who fail to control HIV-1 viremia (Figure1A). In marked contrast, PBMC from non-progressors not only demonstrated virus-specific helper cell responses, but also these responses were of enormous magnitude (Figure 1B). Depletion studies revealed that the effector cells were CD4 positive, and examination of lymphokines triggered for release by these cells demonstrated the cells to be of the Th-1 type, in that IFN gamma but not IL-4 was produced (Rosenberg, Billingsley et al. 1997). Theses cells also secreted antiviral beta-chemokines in response to viral antigen (Cocchi, DeVico et Al. 1995), suggesting a mechanism whereby these cells might further contribute to containment of virus. These results have now been confirmed by a number of additional laboratories, as reported at the 6^th^ Conference on Retroviruses and AIDS in Chicago in February, 1999.

The identification of potent virus-specific helper cell responses in persons who were successfully controlling viremia led us to address the question as to whether these cells were associated with control of viremia in a larger cohort. Ten persons who had never been treated with antiviral therapy were evaluated. In persons with high viral loads there was no evidence of virus-specific helper cell responses to either p4 or gp160, which is also what was observed in both high risk and low risk seronegative persons (Rosenberg, Billingsley et al. 1997). In contrast, in persons who were spontaneously controlling viremia in the absence of antiviral therapy, strong virus-specific helper cell responses were detected (Rosenberg, Billingsley et al. 1997). These same findings were confirmed in a second cohort of infected, untreated patients (unpublished data). These results provide the first evidence of a virus-specific immune response that is associated with control of HIV-1. Recently, the role of CTL in controlling HIV infection has been more firmly established as well. Transient depletion of CD8 cells results in a dramatic increase in viremia in SIV infected macaques (Schmitz, Kuroda et al. 1999; Xin, Bauer et al. 1999). Other studies in humans have shown that adaptively transferred CTL localize at sites of infected cells in vivo (Brodie, Lewinsohn et al. 1999). At least one cross sectional study in humans indicates that there is a negative correlation between CTL and viral load (Ogg, Jin et al. 1998).

Based on our findings of strong Th cell responses in persons who are controlling viremia, and on the detection of strong cytotoxic T lymphocyte (CTL) responses in these persons (Harrer, Harrer et al. 1996), we hypothesize that cellular immunity might be critical to immune regulation in this infection. In response to infection, Th cells are activated to provide help in orchestrating an effective immune response, as one would expect in any viral infection, that is mediated at least in part through CTL. However, because HIV-1 is able to selectively infect activated CD4 cells, we hypothesize that these cells should immediately become infected and deleted. This in turn would lead to insufficient Th cells to maintain CTL responses, and thus CTL responses induced during acute infection would progressively diminish. The progressive loss of CTL is in fact what is observed in the majority of infected persons (Klei8n, van Baalen et al. 1995), consistent with this hypothesis.

If this hypothesis is correct, then acute infection should lead to induction of Th cells in all infected persons. In addition, the immediate institution of antiretroviral therapy at the time these cells are becoming activated should lead to protection of these cells from infection, and in turn lead to maintenance or restoration of these responses. To directly test this, we identified a patient with the acute HIV-1 infection syndrome. He presented with a 2 day history of fever, headache, lymphadenopathy and severe malaise, typical symptoms of this illness (Bollinger, Brookmeyer et al. 1997; Quinn 1997, Kahn and Walker, 1998). An anti-HIV antibody test was negative, but viral RNA in the plasma was greater than 1.2 million copies per ml, establishing a diagnosis of acute HIV-1 infection (Kahn and Walker 1998). He was immediately started on combination antiviral therapy including a protease inhibitor. Viral load promptly dropped to less that detectable, and as this occurred a strong p24-specific proliferative response became detectable (Rosenberg, Billingsley et al. 1997). The same gradual generation of HIV-1 specific Th cell responses to p24 has now been observed in each of the patients treated with antiretroviral therapy during the acute stage of primary HIV-1 infection (Figure 2). Thus early intervention with potent antiviral therapy had restored a virus-specific proliferative response which is similar to that observed in long-term non-progressors (Rosenberg, Billingsley et al. 1997). Further characterization of the breadth and specificity of the response using synthetic peptides to map the targeted epitopes provided further evidence that the responses in these acutely treated patients were of similar quality and quantity to those seen in the non-progressors (data not shown).

These HIV-1 specific Th cell responses typically become detectable within 6-10 weeks of institution of therapy. For all persons studies, the responses to p24 have continued to increase for up to a year of continuous follow-up, despite consistently undetectable viral loads. Several of these persons have also generated gp160-specific Th cell responses as well, but in no case has the magnitude of the gp160-specific response been greater than the p24-specific response.

We now have experience with a subject in whom antiviral therapy was intentionally discontinued according to the initial version of this protocol (98-7242). Virus remained undetectable (<50 copies/ml) for approximately 3 weeks and then slowly rose over the next three weeks to 45,000 copies. The person remained asymptomatic except for an initial pharyngitis that developed after 2 weeks off therapy, which resolved with antibiotics. The discontinuation of therapy was associated with a boost in the magnitude but not breadth of the CTL response, and also a boost in the magnitude of the T helper cell response, which appeared to be transient. The person was asymptomatic at the time that therapy was reinstituted after a total interruption of almost 6 weeks. This person has recently stopped medications again because of a medical indication (hepatitis A infection with markedly elevated liver enzymes). After an initial rise in viral load to 37,700 copies/ml, viral load precipitously declined to 490 copies/ml before drifting up to 6,000 copies/ml. This drop in viral load occurred in the absence of any antiretroviral therapy. Once the patients’ Hepatitis A infection resolved, antiretroviral therapy was restarted. Since the reinstitution of his therapy, we have observed a dramatic boost in HIV-specific T helper cell responses (Figure 3), suggesting that limited viral replication has resulted in a strengthening of HIV-specific immunity. After the first interruption in therapy virus was genotyped and there was no indication of the development of antiviral drug resistance.

1. Rationale and Potential benefits:

These results provide firm evidence that HIV-1 can induce a strong CD4-positive T helper cell response directed against the relatively conserved p24 core protein. In some persons strong responses to the envelope protein gp160 are also generated. Precisely how some individuals are able to generate these responses and become long-term non-progressors is not clear, but may have to do with the relative magnitude of the initial viremia experienced in primary infection. The finding that these responses can be detected in all persons if treated with potent antiviral therapy during acute HIV-1 infection indicates that the induction of these responses is the rule, but that these responses are lost in the early stages of infection in the vast majority of infected persons. The p24-specific proliferative responses correlate with control of viremia (Rosenberg, Billingsley et al. 1997), providing the first evidence that HIV-1 specific immunity is involved in controlling the viral set point. The mechanism of viral inhibition by these cells is likely mediated through CTL, which are able to lyse infected cells before progeny virions are produced (Yang, Kalams et al. 1996). In the acutely treated patients, we have now detected strong HIV-1 specific T helper cell responses in 12 of 12 tested, which are analogous in breadth, magnitude and specificity to those seen in long-term non-progressors.

We hypothesize that early antiviral therapy in acute infection has led to the maturation of an immune response that may be able to contain viremia in infected persons in the absence of ongoing antiviral therapy. This hypothesis is supported by a number of pieces of indirect evidence. First, in a macaque model of lentivirus infection, it has been shown that early treatment with antiviral therapy for 16 weeks is associated with the ability of these animals to control viremia when drug is stopped (Watson, McClure et al. 1997). Of six animals treated in this experimental protocol, five are alive at three years with undetectable viral loads off therapy (N. Haigwood, personal communication). Second, there are two patients treated in the early stages of acute infection who have discontinued therapy and have maintained viral loads below the limits of detection, or at the limits of detection. We have measured HIV-1 specific T helper cell responses in one of these, and have detected stimulation indices of 8-60 on three different occasions during the period off therapy (manuscript submitted). Another reported individual had a transient increase in viral load to 25,000 copies when therapy was stopped, but this decreased to undetectable levels without reinstitution of antiviral therapy (D. Nixon, Oxford University Symposium on long-term non-progressing HIV-1 infection, Oxford England, April 19, 1998). This person had been tested by us before stopping therapy, and had an SI to p24 of 14, consistent with the proposed hypothesis. This same group has now assessed 3 additional persons who have stopped therapy on their own. In total, 2 of 4 have maintained viral loads at the limits of detection off therapy (Ortiz, JCI 1999). .

The ability to control viremia after stopping therapy may not be the case for all persons treated in acute infection, as suggested by a recent report of a person who was treated during acute HV-1 infection and stopped therapy 6 months later, at which time he experienced a recurrence of symptomatic acute HIV-1 infection (Daar, Gai et al. 1998). However, this person had no detectable CTL responses at the time that therapy was stopped, and t helper cell responses were not measured. Thus far all persons that we have treated in the acute stages of infection have maintained viral loads below the limits of detection, and have progressively generated strong CD4 T helper cell responses and the majority (5 of 6 tested thus far) have also generated detectable CTL responses. We propose now to test whether these responses are sufficient to control viremia by interrupting therapy in persons who have developed this immunologic phenotype.

Alternatively, we propose to test whether transient exposure to autologous virus will boost the breadth or magnitude of immunity to HIV-1 such that enhanced immunologic control can be achieved.

Conducting this study has important potential benefits to both the patient and to society. For the individual patient, therapy may be able to be discontinued, diminishing the physical and psychological consequences of the strict multi-drug regimens, and potentially limiting the development of side effects that are becoming increasingly apparent with long-term use of these drugs (Miller, Daly et al. 1998). If viral load is not contained, then allowing a brief period of viral replication may lead to augmentation of existing immune responses when antiviral therapy is re-instituted, such that effective immunity may ultimately be achieved. The benefit to society of these studies is that we may be able to determine the correlates of protective immunity, which would have profound implications for vaccine development and for therapeutic interventions. An additional benefit is that we may be able to determine whether “immunization” with autologous virus is able to boost endogenous immune responses.

1. SPECIFIC AIMS:

We hypothesize that early intervention with potent antiretroviral therapy confers immunologic maturation, which allows for immune control of HIV-1 replication without the need for ongoing drug therapy. Alternatively, we hypothesize that if breakthrough of virus replication is observed, this will provide a boost in HIV-1 specific immunity after reinstitution of antiviral therapy. Primary endpoints will be: (1) time to viral rebound to 200,000 copies/ml and (2) incidence of viral rebound to 200,000 copies/ml. Secondary objectives will be to correlate immunologic and virologic parameters with this beneficial effect including (1) effect on HIV-specific T helper cell responses, (2) effect on HIV-specific CTL responses, and (3) effect on virus genotype. Specifically, we propose to:

1. Discontinue anti-HIV-1 drug therapy in persons treated during the acute infection, who meet the following criteria:
2. Antiviral therapy for at least 4 months, with HIV-1 load of less than <400 RNA copies/ml for at least 2 months, and less than 50 RNA copies/ml prior to stopping therapy.
3. Detectable CD4 T helper cell responses to HIV-1 p24 (delta cpm greater than 500).
4. At least one assay for HIV-1 specific CTL. CTL responses may be detectable or non-detectable.
5. Monitor HIV-1 specific viral load after discontinuation of therapy using an ultra-sensitive RNA quantitation assay (lower limit of detection 50 RNA copies/ml).
6. Reinstitute antiviral therapy when the viral load reaches >200,000 RNA copies/ml, or when it is above 5,000 copies for Re-stop therapy after viral load is suppressed to <50 copies/ml for at least 8 weeks. Parameters for re-starting therapy will be used as above. Each subject will have therapy stopped a maximum of four times.
7. Virus will be genotyped each time it returns to ensure that resistance is not developing. If resistance does develop, then the patient will be informed and an alternative regimen suggested to which the virus is predicted to be sensitive, based on genotype. In persons who develop drug resistance, no further discontinuation of therapy will be pursued.
8. Summary of parameters to be monitored:
9. Time to recurrence of viral load> 200,000 copies
10. Incidence of recurrence of viral load > 200,000 copies
11. Effect on CTL
12. Effect on T helper cell responses
13. Effect on virus genotype
14. Effect on CD4 count
15. SUBJECT SELECTION:
16. Inclusion criteria:

1. CD4 proliferative responses in fresh PBMC of greater than 500 cpm to HIV-1 p24, using the standard assay (Rosenberg, Billingsley et al. 1997).

2. Antiviral drug therapy, three drugs, instituted during seroconversion, as defined by an indeterminate Western blot, or detuned ELISA or the clinical picture3.An HIV-1 load for at 2 months of <400 RNA copies/ml.

1. An HIV-1 load at the time of entry into the study of less than 50 RNA copies/ml (i.e., below the limits of detection by the ultra-sensitive assay).
2. Ability to give informed consent.
3. CD4 cell count within the normal range as per MGH hematology laboratory parameters.
4. Minimum Hgb of 11 gm/dl
5. Exclusion criteria:
6. Persons who do not meet the inclusion criteria.
7. Pregnant women or women intending to get pregnant during the time of the trial.
8. Age less than 18 years.
9. HIV-1 related opportunistic infections.
10. Other illness that would interfere with interpretation of the data, such as immunologic disorders or disorders requiring the use of immunomodulatory agents.
11. Vaccinations within 4 weeks prior to study entry.
12. Restarting therapy including Abacavir due to risk of hypersensitivity reaction.
13. Evidence of HIV drug resistance, as shown by the presence of major, well known drug mutations.

Source of subjects: Subjects will be recruited from an existing cohort of persons with documented acute HIV-1 infection who were treated with potent antiviral therapy. Persons have been followed at MGH in the Outpatient ID Clinic, referred by their primary care physician, or other outpatient clinics of the MGH, or other health care facilities.

1. SUBJECT ENROLLEMENT:
2. Methods of enrollment: Persons will be enrolled from an existing cohort of persons who have been treated with acute HIV-1 infection.
3. Procedures for obtaining informed consent: Informed consent will be obtained by the PI or Co-PI. Consent will be obtained prior to stopping therapy after extensive discussion.
4. Treatment assignment/randomization: N/A.
5. STUDY PROCEDURES:
6. Study visits and parameters to be measured: After the initial screening and enrollment visits, persons will be seen at least for the first 24 weeks, and then monthly. With a total of 30 visits for the first year. In Year 2, visits continue monthly, for a total of at least 12 visits. The following parameters will be measured:
7. Time to viral rebound: Virus load by ultra-sensitive PCR assay (Roche) will be determined at each of the follow-up times, with results available within 48-72 hours. Time to viral rebound and the rate of rise of viremia will be measured. Additionally, patients may be immediately evaluated by a study physician for any inter-current illness. This requires 1 ml of plasma.
8. CD4 proliferative responses: CD4 proliferative responses to p24 and gp160 will be performed each week for the first 8 weeks, and then every two weeks. This requires 15 ml of blood.
9. CTL precursor frequency assays (CTLp): CTLp will be measured every other week, to Gag, Pol, Env and Nef, using standard cytotoxicity assays. This requires <5 ml of blood.
10. CD4 cell counts; CD4 cell counts will be obtained once a week for the first four weeks, then every other week for the next 20 weeks, and then monthly. This requires 3 ml of blood.
11. If persons present with a febrile illness, the investigators may choose to perform the following additional tests or refer the person to the PCP:

# Influenza Titer

Mono spot

Strep test

Serologies for other common viral illness

This will help assess if the febrile illness is related to the study or not.

Estimation of blood volume required for these assays: 25 ml of blood will be required pre-enrollment for baseline assays. For the first 8 weeks, up to 75 cc’s of blood will be drawn weekly. For the next 16 weeks, 60 cc’s of blood will be drawn per week. A total of 600 cc’s will be taken for the first 8 weeks, 960 cc over the next 16 weeks, and up to 360 cc over the next 6 months. In years two through seven, up to 960 cc’s of blood will be drawn over the course of the year.

Contact with study subjects: Drs. Walker, Rosenberg, Robbins, or the study nurse will speak with each subject by phone frequently during the duration of the trial. Dr. Rosenberg will be available by beeper 24 hours a day. Subjects and their health care providers will have ongoing real-time access to the clinically relevant parameters and will discuss patient management issues with the PI on a regular basis.

1. Drugs to be used; N/A.
2. Devices to be used; N/A.
3. Procedures/surgical interventions: N/A.
4. Data to be collected and when data are to be collected: The data to be collected are outlined in section A above. The data will be collected at each visit.
5. Reinstitution of therapy: Therapy will be re-instituted for any single viral load of greater than 200,000 RNA copies per ml of plasma. If a plateau of viremia of greater than 5,000 RNA copies per ml of plasma is achieved, then antiviral therapy will be reinstituted. The same antiviral therapy that had been taken at the time of enrollment into this study will be re-instituted, excluding Abacavir which can not be restarted. unless there is evidence of resistance by genotyping. Therapy will be re-instituted for any sustained CD4 cell count decrease of 25% from baseline. Persons will subsequently be monitored on a weekly basis with viral load, CD4 T helper cell responses, and CTL assays to determine whether viremia is suppressed and whether an augmentation of HIV-1 specific immunity is observed. Persons will subsequently be monitored on a weekly basis with viral load, CD4 T helper cell responses, and CTL assays to determine whether viremia is suppressed and whether an augmentation of HIV-1 specific immunity is observed. CD4 cell counts are measured every other week.

After restarting therapy, the visit schedule is weekly until the HIV RNA is <50 copies per ml plasma. The viral load <50 must be sustained for at least an eight week period, when persons will once again be given the opportunity to stop therapy. At this point, if persons elect not to stop therapy, the visit schedule changes to onece a month, and the option of stopping therapy remains open if the viral load continues to be <50 copies/ml.

1. Re-stopping of therapy: In persons who experience a rebound in Viral Load after stopping therapy, drugs will be restarted as above (F) and subjects will be monitored. After 8 weeks of undetectable vial load (<50 RNA molecules per ml) they will be given the option of stopping therapy a second time under the same conditions of evaluation as occurred after the first interruption in therapy. Therapy will be re-instituted using the same criteria as in (F) above. Patients will be treated for at least 8 weeks with viral load less than 50 copies when they will once again be given the option to stop therapy, as in (F) above. The cycle can continue for a maximum of four interruptions.
2. Early study termination: This study will be discontinued in individuals noted to develop significant antiviral genotype resistance. Any subject with evidence of new mutations in bulk PCR product, not present in pre-treatment baseline plasma RNA (suggesting evolution of antiretroviral resistance) will result in termination of study and no further attempts to discontinue therapy will be done.
3. If a person chooses to go against the protocol criteria and guidelines to stop or start therapy on their own, they may continue on an observational follow up status. Their care would be directed by their Primary Care Physician and not the study guidelines.

As a suggestion of the IRB comments on 5/6/03: Clarification of follow up to read:

Long term follow up of these patients consists of clinic visits every 6 weeks, in which safety parameters, i.e., CD4 and viral load are performed in addition to experimental immunologic assays.

1. BIOSTATISTICAL ANALYSIS:
2. Specific data variables being collected for the study:
3. Viral load
4. T helper cell responses
5. CTL precursor frequency
6. CD4 cell counts
7. Times to viral rebound and rate of viral rebound
8. Viral genotype for resistance mutations
9. Study endpoints: See attached schema. The endpoint will be the time to rise in viral load to greater than 200,000 RNA copies/ml plasma using the Roche Ultra-Direct assay. At that point the study subject will restart antiretroviral therapy. Alternatively, if a plateau of viremia of greater than 5,000 RNA copies per ml of plasma is achieved, then antiviral therapy will be re-instituted. Therapy will consist of those drugs, which had been fully suppressive prior to stopping therapy, with the exception of Abacavir which cannot be reintroduced after stopping therapy.

C. Statistical methods:. Discontinuation of therapy in chronic infection is associated with a rapid rebound in viremia. We will measure the time to viral rebound and determine whether this increases with subsequent discontinuation of therapy. We will also look for changes in HIV-1 specific immune function over the course of study, and will compare this to any changes seen in persons with continued therapy. Reproducible changes in virus-specific immune function of greater than 5 fold will be considered significant, based on the observed intra-sample variability of each of these tests, which is less than 2-fold (data not shown). We will also measure the rate of rise of viremia should it recur, and calculate whether the rate rises more slowly with subsequent stops in therapy.

D. Power analysis: N/A.

1. RISKS AND DISCOMFORTS:

The risk involved are those related to uncontrolled virus replication in the setting of HIV-1 infection, and the possibility of the emergence of drug resistant virus. Emerging data now demonstrate that simultaneous discontinuation of antiviral therapy in HIV-1 infection results in the replication of wild-type, drug sensitive virus, and that reinstitution of therapy results in the prompt inhibition of replication (Avidan Neuman, personal communication and manuscript in press; Marty Markowitz, personal communication).

Thus other studies have already shown that there is not an appreciable risk in this regard. Patients will be monitored for genotypic resistance each time virus rebounds, and will not undergo additional rounds to stopping therapy if resistance is present.

Another risk involves the potential for increased seeding of a population of latently infected cells. Recent studies have demonstrated that this pool is stable regardless of disease stage and that eradication of this pool is not being achieved with prolonged antiviral therapy (Finzi, Hermankova et al. 1997). In addition, in the one person who has been studied in whom therapy was halted, the size of this pool remained comparable to the pool size in persons remaining on therapy. However, it is possible that this patient was the exception and that the reservoir of latently infected cells will increase if therapy is interrupted. This will be directly monitored by obtaining baseline measurements of the size of the pool in persons pre-interruption of therapy, and following reinstitution of therapy.

A third risk is related to venipuncture.

A fourth risk relates to the possibility for anxiety related to stopping drug therapy.

A fifth risk involves the possible recurrence of an acute infection syndrome if virus replication recurs after stopping therapy. At least one such case has been reported (Daar, Bai et al. 1998), but this person had no CTL responses at the time therapy was stopped at six months. In addition, CD4 proliferative responses were not assessed in that patient.

A sixth risk is that CD4 count will drop and not rise again with reinstitution of antiviral therapy.

1. POTENTIAL BENEFITS:

The potential benefit to the patient is that we may determine that drug therapy is not required but that the immune system can contain the level of viremia below or near the limit of detection. Other potential benefits will be to society rather than to the individual patient. These studies will allow us to determine whether the immune responses, which have been generated in these individuals, are sufficient to control viremia, which has important implications for vaccine development and for the development of immunotherapies against HIV.

1. MONITORING AND QUALITY ASSURANCE:
2. Independent monitoring of source data: An internal independent monitoring board will be used to review protocol compliance and ensure that the trial is being conducted according to the established guidelines. This board will meet annually, and consists of Dr. Martin Hirsch and Dr. Benjamin Davis. The PI will present updates.
3. Safety monitoring: Patients will be seen in follow-up in the outpatient clinics at MGH, where they will be monitored for adverse effects of stopping therapy. These include symptomatic illness related to reemergence of viremia, and the development of antiviral drug resistance. Antiviral therapy will be re-instituted for recurrence of symptoms of acute infection associated with viral loads of greater than 200,000 RNA copies per ml. Drug therapy will be altered based on viral genotype, and therapy will not be discontinued again in persons who develop significant genotypic resistance. Should symptomatic illness occur at lower viral loads, then the subject will be given the option of restarting therapy.
4. Outcomes monitoring: Outcomes will be monitored by the PI, and presented to the internal review panel on a annual basis.
5. Adverse events reporting guidelines: Adverse events related to this study will be reported to by the PI to the independent monitoring board (see A above) and will be reported to the human studies committee. This trial is unlike most trials because we are not testing a new intervention but are determining whether drug therapy can be halted. Therefore adverse events will be those associated with the reemergence of viremia, which potentially consist of fever, rash, headache, aseptic meningitis, pharyngitis, myalgia, gastrointestinal distress, oral ulcers, lymphadenopathy (Kahn and Walker 1998). These events will be reported by the PI to the human studies committee and to the internal review board. Adverse events will be scored as probably, possibly, remotely or unrelated to the interruption in therapy.

AMENDMENTS:

Amendment 1: Submitted :3/8/00 – increase subjects from 12 to20

Approved: 3/29/00

(Title of protocol at this time was: Therapeutic immunization in

HIV infection by controlled interruption of therapy)

Amendment 2: ?

Amendment 3: This should be amendment # 2

Submitted: 8/28/00 –

Consent form change page 2. Under procedures, first paragraph. Antiretroviral medications must be taken for a least 6 months. Viral load <400 copies/ml over the last 4 months.

Update protocol 7/18/00 pages 8A. #2. Removed “one of which is a protease inhibitor”.

Page 9 #2 has been deleted

Page 9 #4 should read 20 weeks, not 12

Approved as Amendment # 3 on 9/6/00

Amendment 3: Submitted

Increase total # of subjects to 50

Title change to represent age range to 18-64 or older

Amendment 4: Submitted with 2002 continuing review

Change PI from Bruce Walker to Eric Rosenberg

See attached

Amendment 5: Submitted with 2003 continuing review

Add: Melinda Boczanowski, M.A. as study staff

Amendment 6: As a suggestion of the IRB comments on 5/6/03:

Clarification of follow up to read:

Long term follow up of these patients consists of clinic visits every 6 weeks, in which safety parameters, i.e., CD4 and viral load are performed in addition to experimental immunologic assays.

Amendment 7: Remove as study staff as they no longer work at MGH: Philip Norris, M.D., Kristen Eutizzi, RN., Melinda Boczanowski
