# Supplementary material for: Limited Durability of Viral Control following Treated Acute HIV Infection
Source: PLoS Med. 2004 Oct 26;1(2):e36. doi: 10.1371/journal.pmed.0010036 (PMC524377; doi:10.1371/journal.pmed.0010036)
Supplement: Protocol S2 — (47 KB PDF). [file pmed.0010036.sd002.pdf]

RECEIVED
AUG 26 1999
DIVISION OF RESEARCH AFFAIRS

AMENDMENT FORM

Use this form for any amendment(s) to your existing approved and ongoing protocol
(This includes additions, deletions, exceptions to enrollment, protocol revisions, etc.).

Principal/Overall Investigator: Bruce D. Walker	Phone Number: 617-724-8332
Study Title: Therapeutic immunization in HIV infection by controlled interruption of therapy	
Protocol#: 99-7178	Protocol Update/Amendment#: 1

Please complete the table below. The table is designed for you to indicate which component(s) or element(s) of your protocol are being changed. **Please check all applicable items.**

Component(s) or Protocol Element(s) to be Changed

√		√	
☐	Investigators (P.I./Overall/Site Responsible)	☐	Study Type
☐	Co-Investigator(s)	☐	Recruitment Procedures
☐	Project Staff	☐	Consent Procedures
☐	Protocol Title	☐	Duration of Subject Participation
☐	Sponsor	☐	Patient Diaries
☐	Keywords	☐	Questionnaires
☐	Version	☐	Remuneration
☒	Site Enrollment Number of Subjects	☐	Research Related Use of Medical Records
☒	Total Enrollment Number of Subjects	☐	Research Related Use of Discarded Material
☐	Types of Subjects	☐	Use of Specimens
☐	Source of Subjects	☐	Consent Form(s)
☐	Site Utilization	☐	Other

Protocol amendments frequently require revising documents used to guide and/or recruit subjects. Please consider whether your amendment(s) require(s) the submission of any supporting documentation and indicate below all documents appended.

☐	Revised Protocol (New Version)	☐	Sponsor Letter With Description of Changes
☐	Revised Consent Form	☐	Revised Advertisement
☐	Other (please describe)	☒	PI Memo Describing Changes

Please fully describe your amendment(s) below*. List the current item followed by the new or revised item or a redline version of the changed document. End with the reason or justification for the change (e.g., eligibility age range is 30 to 50 yrs.; wish to revise to 30 to 60 yrs. Because insufficient numbers being recruited). Include a discussion of how the change might affect the integrity of the protocol and the risk/benefit ratio. (The more complex the change and reasons for the change, the more information likely needed for review).

***Amendment(s):** Increase subjects from12 to 20 site-wide and total, as more subjects have been identified since inception of protocol.

[signature]
Principal/Overall Investigator's Signature

8/20/99
Date
