# Supplementary material for: Limited Durability of Viral Control following Treated Acute HIV Infection
Source: PLoS Med. 2004 Oct 26;1(2):e36. doi: 10.1371/journal.pmed.0010036 (PMC524377; doi:10.1371/journal.pmed.0010036)
Supplement: Protocol S3 — (187 KB PDF). [file pmed.0010036.sd003.pdf]

Research Consent Form

Brigham and Women's Hospital
Dana-Farber Cancer Institute
Massachusetts General Hospital

Version III.a August 1998
Pi_dist9.doc

Imprint Patient ID Number

Protocol Title: Structured Treatment Interruption in Persons with Acute HIV-1 Infection

Principal/Overall Investigator: Eric Rosenberg, MD

Site-Responsible Investigator(s)/Institution:

Co-Investigator(s)/Study Staff: Bruce Walker, MD, Gregory Robbins, MD, Philip Norris, MD, Marcus Altfeld, MD, Marylyn Addo, MD, Mary Johnston, RN, Colleen Corcoran, NP, and Kristen Eutizzi, RN

Description of Subject Population: Persons with HIV/AIDS

PURPOSE

We would like permission to enroll you as a participant in a research study. The purpose of the study is to evaluate the effects of early treatment of acute (very recent) HIV-1 infection on the ability of the immune system to control this infection in the absence of ongoing drug therapy. You are being asked to participate in this study because you are a person who was diagnosed with acute HIV infection and started taking anti-HIV medications at that time.

HIV infection appears to prevent the immune system (cells and proteins in the body which fight viral infections) from functioning normally. However, some persons have been infected for 20 years, have never been treated with antiretroviral (anti-HIV) drugs, and have never developed AIDS or evidence of immune system damage. These persons, who are often called long-term non-progressors, have particularly strong HIV specific immune responses to the virus. Early treatment of people with acute HIV-1 infection may encourage the development of immune responses that are similar to those of long-term non-progressors. It is hoped that early treatment with antiretroviral medicine, and the development of strong HIV specific immune responses to the virus, will allow persons to control the virus without the need for ongoing drug treatment. In this research study, we hope to determine whether persons treated with antiretroviral therapy in the early stages of the HIV infection can later control the virus without drug therapy due to this immune response. If virus is not controlled, we will determine whether restarting antiretroviral therapy and then stopping antiretroviral therapy again will boost the HIV specific immune response to the virus. The only medications that will be stopped as part of this study will be those that are anti-HIV medications. If you are on medications to treat other conditions, you will continue taking these as your health care provider prescribed.

Approximately 50 subjects will be enrolled at the Massachusetts General Hospital. The duration of the study is 7 years, but you have the right to discontinue your participation at any time.

STUDY CONTACTS

You are urged to discuss any questions you may have about this study with the study staff. The Principal Investigator of this study is Dr. Eric Rosenberg, and he can be reached through the MGH page operator at 617-726-2241 twenty-four hours a day. Other study staff include Dr. Bruce Walker, who can be reached at (617)724-8332, and Dr. Greg Robbins, Mary Johnston, RN, and Colleen Corcoran, NP,who can also be reached through the MGH page operator.

PROCEDURES

Before beginning your participation in this study, your study nurse or doctor will review your chart to make sure you are eligible. In order to participate in this study, you must be infected with HIV and you need to have started taking antiretroviral medications within 180 days of seroconversion (seroconversion is when your HIV test turns from negative to positive.) You must have taken antiretroviral medications for at least 4 months, and your HIV viral load needs to be less than 400 copies/ml over the last 2 months, and less than 50 copies/ml at the time you start the study. In addition, researchers will check to make sure you have good HIV specific immune responses (how well your immune system can fight HIV.)

You cannot participate in this study if you currently have an infection, or if you have a disease that involves the immune system, e.g. rheumatoid arthritis, sarcoidosis, or lupus. Also, if you use any medications that affect your immune system, such as steroids, Epogen, G-CSF, or Interferon, you will not be able to enroll in the study.

You will have a research appointment at the clinic to see if you are eligible for the study. Blood will be collected for CD4 count, plasma HIV viral load, and immunological assays (research laboratory tests to determine how well your immune system responds to HIV.) If you are a female of childbearing potential, a pregnancy test will be performed, and you will not be allowed to participate in this trial if you are pregnant or nursing, or if you are trying to get pregnant. If you enroll in this study, you will be asked to use birth control to prevent pregnancy.

If you meet the eligibility requirements, and you choose to participate, you will come back for another research visit within 30 days. At this visit, you will be instructed by the study nurse or doctor on how and when to stop your HIV medications. Your participation in this study will be discussed with your primary care provider, and s/he will be informed about the results of the clinical monitoring lab tests that will be performed over the course of the study.

Once you stop taking your antiretrovirals, you will be asked to come back into the clinic for weekly visits for the first 24 weeks, and then every month for the remainder of your participation in the study (unless you start taking antivirals and then stop again some point in the future.) For the first 8 weeks, 75cc's (5 tablespoons) of blood will be drawn at each visit, which means a total of 600cc (2 and ½ cups) of blood, which is a little more than a standard blood donation, will be drawn over 8 weeks. For the next 16 weeks, 60cc's (4 tablespoons) of blood will be drawn at each visit, which is 960cc (4 cups) over 16 weeks. Up to 360 cc (1 and ½) cups) of blood will be drawn over the next 6 months. After the first year in the study, up to 960 cc (4 cups) will be drawn over the course of each following year.

**Subject Population:**	Persons With HIV/AIDS		
**IRB Protocol Number:**	2000P-000341	**Sponsor Protocol Number:**	N/A
**Consent Form Valid Date:**	June 7, 2002	**Amendment Number Approved:**	HRC #4
**IRB Expiration Date:**	May 23, 2003	**Amendment Approval Date:**	May 23, 2002

At these visits, your bloods will be tested for CD4 measurements, a complete blood count (CBC), viral load measurements and research immunology assays. A symptom directed physical examination will be performed which may include assessments of skin, ear, nose, throat, lymph nodes, and any other body systems which are the focus of a complaint, and study staff will you record any medications you are taking.

Antiretroviral therapy will be started again if your blood tests reveal **any** of the following:

1.) You have a single HIV viral load greater than 50,000 copies/ml **OR**
2.) You have HIV viral loads that are greater than 5,000 copies/ml for three consecutive visits **OR**
3.) You have a sustained (seen at multiple time points) 25% or greater decrease in your CD4 count

The same antiretroviral therapy that was taken at the time of enrollment of the study will be re-instituted; however, if you were taking abacavir (Ziagen) before you stopped your medications, this drug will not be restarted, because restarting this medication would put you at risk for having an allergic reaction. Also, your antiretroviral medications may be changed if there is evidence that you have developed drug resistance. We can test for drug resistance through genotyping (a test that shows us what antiviral medications you may be resistant to).

After beginning drug therapy again, you will be asked to come in on a weekly basis for blood tests. The blood tests will include HIV viral load to determine whether your virus has been suppressed and research immunology assays to determine if there has been a boost to your HIV-specific immune response. Complete blood cell count and CD4 measurements are checked every other visit. You will be asked to come in every week until your HIV viral load is undetectable again (less than 50 copies/ml).

If your HIV viral load remains less than 50 copies/ml for 8 weeks, you will be given the opportunity to stop taking the antiretroviral drugs again. If you choose not to stop therapy, you will be asked to come in once a month to monitor your viral load and perform research immunology assays. The opportunity to stop therapy will remain open as long as the viral load remains under 50 copies.

If you choose to stop therapy, you will be advised again on how and when to stop your antiretroviral medications. You will be asked to come in for weekly visits for the first 24 weeks and then monthly after that, following the same appointment schedule as the first time you stopped taking the medicine. If your blood tests reveal that your HIV viral load has risen or your CD4 count has dropped by the guidelines outlined in this consent, then you will be asked to re-start the drug therapy under the protocol. You will be able to cycle from taking drug therapy to not taking drug therapy up to four times over seven years.

If at any point, your blood tests reveal that you have developed major resistance to the medications, the study will be stopped, and there will be no further interruptions in your HIV drug therapy.

**Subject Population:** Persons With HIV/AIDS			
**IRB Protocol Number:**	2000P-000341	**Sponsor Protocol Number:**	N/A
**Consent Form Valid Date:**	June 7, 2002	**Amendment Number Approved:**	HRC #4
**IRB Expiration Date:**	May 23, 2003	**Amendment Approval Date:**	May 23, 2002

Your blood obtained as part of this study will be stored using a code to protect your identity, and we would like your permission to use it for AIDS-related research in the future. The link between the code and your name will be kept in the research nurse's office in a locked file cabinet, and only study staff will have access to this information. An immortalized cell line will be established using your white cells (B lymphocytes) and this cell line will be used to assist in measuring your immune function. This means an endless supply of your blood cells will be available for research.

Do you agree to having your blood immortalized for future used in AIDS-related research?

Yes ______________ No ________________ Please initial: ________________

Do you agree to have frozen samples of your blood, identified only by a code, stored indefinitely and used in the future for other HIV-related research?

Yes ______________ No ________________ Please initial: ________________

You may be reimbursed up to $20 per visit for parking expenses.

COSTS
Neither your insurance company nor you will be charged for any procedure involved with this study. You will not be charged for the clinic visits, tests or evaluations associated with your participating in this study. However, you or your insurance carrier will continue to be responsible for your regular medical expenses, including your antiretroviral medication.

RISKS AND DISCOMFORTS
A potential risk of stopping antiretroviral drug therapy is that the virus will become resistant to the drugs you have been taking. This is not likely to occur, because other research has shown that stopping all drugs at once does not lead to the development of resistance, and your viral load will be closely monitored so that drug therapy will be restarted at the appropriate time. Nevertheless, it is possible that your virus will become less inhibited by the drug cocktail, and that an alternative drug regimen may be needed. You will be monitored closely for the development of resistance.

Another potential risk is the infected presence of a population of infected cells which do not actively produce virus in the blood ("latent pool") may increase. However, other studies have shown that this pool of latently infected cells remains stable on antiretroviral therapy, but it is unknown if this pool will get any larger with treatment interruption. The pool of latently infected cells in your body will be monitored over the course of this study.

**Subject Population:**	Persons With HIV/AIDS		
**IRB Protocol Number:**	2000P-000341	**Sponsor Protocol Number:**	N/A
**Consent Form Valid Date:**	June 7, 2002	**Amendment Number Approved:**	HRC #4
**IRB Expiration Date:**	May 23, 2003	**Amendment Approval Date:**	May 23, 2002

It is also possible that re-exposure to virus will cause a decrease in CD4 counts, and that these numbers may not increase with the reinstitution of therapy. Again, you will be restarted on medication if your CD4 count drops 25%, and your CD4 count will be followed closely throughout the course of the study.

You may experience anxiety over stopping HIV medications. You may also experience a possible reoccurrence of a flu-like syndrome (fevers, sore throat swollen glands, muscle aches and pains, headache and rash) if the amount of virus in your blood begins to increase. Study staff will watch you closely for this.

Sampling blood may cause a brief amount of pain, bleeding, or temporary bruising. Rarely, a person feels faint when their blood is drawn. In extremely rare instances, an infection develops at the needle insertion site.

BENEFITS

The potential benefit to you is that you **may** be able to stop drug treatment for HIV infection. The potential benefit to society is that researchers may gain a better understanding of how the body's natural defenses fight HIV, which may help in the development of a vaccine.

ALTERNATIVES

The alternatives to participating this study is to continue on your present treatment regimen. If you choose not to participate in this study, your care at MGH will not be affected in any way.

If you have recently decided to stop your antiretroviral medications with or without instruction from your primary care provider, you may join this study on an observational basis only, which means that you are not following the research protocol. You will be asked to come in on a regular basis and we will study your immune system for HIV-specific responses, and monitor your HIV viral load level and CD4 counts. As an observational participant in this study, we will provide you and your primary care provider with clinical lab results. However, the decision of if or when you restart your antiretroviral medications will be decided by you and your provider, and not dictated by study guidelines.

Conversely, if at any point during the study you decide to not restart your HIV medications despite the protocol guidelines and the recommendations of the study team, your participation in the study would change to an observational basis. You will be asked to come in on a regular basis so we can continue to study your immune system for HIV-specific responses and monitor your HIV viral load and CD4. As an observational participant in this study, we will continue to provide you and your primary care provider with clinical lab results. **All future decisions regarding starting or stopping HIV medications would no longer be dictated by the protocol guidelines and would be determined by you and your provider.**

Do you agree to continue/participate in this study as an observational participant?

Yes ______________ No ______________ Please initial: ______________

THE FOLLOWING PARAGRAPHS CONTAIN STANDARD INFORMATION WHICH GENERALLY APPLIES TO PERSONS INVOLVED IN A RESEARCH STUDY AND ARE REQUIRED ON ALL CONSENT FORMS.

CONFIDENTIALITY

Medical information produced by this study will become part of your hospital medical record, unless specifically stated otherwise in this consent form. Information that does not become part of your medical record will be stored in the investigator's file and identified by a code number only. The code key connecting your name to specific information about you will be kept in a separate, secure location. Your medical record is available to health care professionals at Brigham and Women's Hospital (BWH), Dana-Farber Cancer Institute (DFCI), or Massachusetts General Hospital (MGH), collectively called the "Hospitals", and may be reviewed by appropriate Hospital staff members in the course of carrying out their duties; however, they are required to maintain confidentiality in accordance with applicable laws and the policies of the Hospitals. Information contained in your records may not be given to anyone unaffiliated with the Hospitals in a form that could identify you without your written consent, except as described in this consent form or as required by law.

It is possible that your medical and research record, including sensitive information and/or identifying information, may be inspected and/or copied by the study sponsor (and/or its agent), the Food and Drug Administration (FDA), federal or state government agencies, or hospital accrediting agencies, in the course of carrying out their duties. If your record is inspected or copied by the study sponsor (and/or its agents), or by any of these agencies, the Hospitals will use reasonable efforts to protect your privacy and the confidentiality of your medical information.

The results of this study may be published in a medical book or journal or used for teaching purposes. However, your name or other identifiers will not be used in any publication or teaching materials without your specific permission. In addition, if photographs, audiotapes or videotapes were taken during the study that could identify you, then you must give special written permission for their use. In that case, you will be given the opportunity to view or listen, as applicable, to the photographs, audiotapes or videotapes before you give your permission for their use if you so request.

REQUEST FOR MORE INFORMATION

You may ask more questions about the study at any time. The investigator(s) will provide their telephone number so that they are available to answer your questions or concerns about the study. You will be informed of any significant new findings discovered during the course of this study that might influence your continued participation.

If during the study or later, you wish to discuss your rights as a research subject, your participation in the study and/or concerns about the study, a research-related injury with someone not directly involved in the study, or if you feel under any pressure to enroll in this study or to continue to participate in this study, you are asked to contact a representative of the Human Research Committees at BWH (617) 525-3170, at MGH (617) 726-3494, or at the Protocol Administration Office at DFCI (617) 632-3029. A copy of this consent form will be given to you to keep.

Imprint Patient ID Number

REFUSAL OR WITHDRAWAL OF PARTICIPATION

Participation in this study is voluntary. You do not have to participate in this study. Your present or future care will not be affected should you choose not to participate. If you decide to participate, you can change your mind and drop out of the study at any time without affecting your present or future care in the Hospitals. In addition, the doctor in charge of this study may decide to end your participation in this study at any time after he/she has explained the reasons for doing so and has helped arrange for your continued care by your own doctor, if needed.

INJURY STATEMENT

If you are injured during the course of the study and as a direct result of this study, you should contact the investigator at the number provided. You will be offered the necessary care to treat that injury. This care does not imply any fault or wrong-doing on the part of the Hospitals or the doctor(s) involved. Where applicable, the Hospitals reserve the right to bill third party payers for services you receive for the injury. The Hospitals will not provide you with any additional compensation for such injuries.

SIGNATURE

I confirm that the purpose of the research, the study procedures and the possible risks and discomforts as well as potential benefits that I may experience have been explained to me. Alternatives to my participation in the study also have been discussed. All my questions have been answered. I have read this consent form. My signature below indicates my willingness to participate in this study.

______________________________ ______________
Subject Date

______________________________ ______________
Witness/Advocate/Minor/Legal Guardian (if required) Date

______________________________ ______________
Additional Signature (if required)(identify relationship to subject) Date

I have explained the purpose of the research, the study procedures, identifying those that are investigational, the possible risks and discomforts as well as potential benefits and have answered any questions regarding the study to the best of my ability.

______________________________ ______________
Study Representative Date

Investigator Complete This Information At Enrollment for HEMATOLOGY/ONCOLOGY PROTOCOLS ONLY
(A copy of this form should be faxed to the QCC and to the appropriate Medical Records)

Primary Care Physician's Name:
Date protocol treatment begins for this subject:
Diagnosis:

Subject Population: Persons With HIV/AIDS

**IRB Protocol Number:**	2000P-000341	**Sponsor Protocol Number:**	N/A
**Consent Form Valid Date:**	June 7, 2002	**Amendment Number Approved:**	HRC #4
**IRB Expiration Date:**	May 23, 2003	**Amendment Approval Date:**	May 23, 2002
