# Supplementary material for: Limited Durability of Viral Control following Treated Acute HIV Infection
Source: PLoS Med. 2004 Oct 26;1(2):e36. doi: 10.1371/journal.pmed.0010036 (PMC524377; doi:10.1371/journal.pmed.0010036)
Supplement: Protocol S4 — (36 KB PDF). [file pmed.0010036.sd004.pdf]

PARTNERS HEALTHCARE SYSTEM

Human Research Committee
Massachusetts General Hospital
Lawrence House
10 North Grove Street
Boston, MA 02114
(617) 726-3494

Continuing Review: Notification of IRB Approval/Activation

Protocol #: 2000-P-000341/8; MGH Legacy #: 99-7178

Date: 06/06/2003

To: Eric Rosenberg, MD
Medical Services 30AA
GRJ 504

From: Ruth V. Benjamin
BWH Administration 72AA
10 Brookline

Title of Protocol:	Structured Therapy Interruption In Persons With Acute HIV-1 Infection
Sponsor:	MGH Departmental Funds
IRB Continuing Review #:	4
IRB Review Type:	Full
IRB Approval Date:	05/06/2003
Approval Effective Date:	06/06/2003
IRB Expiration Date:	05/06/2004

This Project has been reviewed and approved by the MGH IRB, Assurance # FWA00003136. During the review of this Project, the IRB specifically considered (i) the risks and anticipated benefits, if any, to subjects; (ii) the selection of subjects; (iii) the procedures for securing and documenting informed consent; (iv) the safety of subjects; and (v) the privacy of subjects and confidentiality of the data.

Please note that if an IRB member had a conflict of interest with regard to the review of this project, that member left the room during the discussion and the vote on this project.

NOTE: Closed to enrollment as of February 2003. Treatment and/or active follow up continues.

As Principal Investigator you are responsible for the following:

1. Submission in writing of any and all changes to this project (e.g., protocol, recruitment materials, consent form, etc.) to the IRB for review and approval prior to initiation of the change(s), except where necessary to eliminate apparent immediate hazards to the subject(s). Changes made to eliminate apparent immediate hazards to subjects must be reported to the IRB within 24 hours.
2. Submission in writing of any and all adverse event(s) that occur during the course of this project that are both serious and unexpected within 10 working/14 calendar days of notification of event.
3. Use of only IRB approved copies of the consent form(s), questionnaire(s), letter(s), advertisement(s), etc. in your research. Do not use expired consent forms.
4. Informing all physicians listed on the project of changes and adverse events.

The IRB can and will terminate projects that are not in compliance with these requirements. Direct questions, correspondence and forms (e.g., continuing reviews, amendments, adverse events, safety reports) to Ruth Benjamin, (617) 525-3191.

cc: Colleen P Corcoran, RN, Medical Services 30AA, FND 8
Ellen Kornell, Medical Services 30AA, CNY 149 5022

MASSACHUSETTS GENERAL HOSPITAL

PARTNERS HEALTHCARE SYSTEM

Human Research Committee
Massachusetts General Hospital
Lawrence House
10 North Grove Street
Boston, MA 02114
(617) 726-3494

Continuing Review: Notification of IRB Approval/Activation

Protocol #: 2000-P-000341/9; MGH Legacy #: 99-7178

Date: 04/13/2004

To: Eric Rosenberg, MD
Medical Services
GRJ 504

From: Ruth V. Benjamin
BWH Administration
10 Brookline Place West

Title of Protocol:	Structured Therapy Interruption In Persons With Acute HIV-1 Infection
Sponsor:	MGH Departmental Funds
IRB Continuing Review #:	5
IRB Review Type:	Expedited
IRB Approval Date:	04/12/2004
Approval Effective Date:	04/13/2004
IRB Expiration Date:	04/12/2005

This Project has been reviewed and approved by the MGH IRB, Assurance # FWA00003136. During the review of this Project, the IRB specifically considered (i) the risks and anticipated benefits, if any, to subjects; (ii) the selection of subjects; (iii) the procedures for securing and documenting informed consent; (iv) the safety of subjects; and (v) the privacy of subjects and confidentiality of the data.

NOTE: Findings noted. Closed to enrollment as of February 2003. Data analysis only.

As Principal Investigator you are responsible for the following:

1. Submission in writing of any and all changes to this project (e.g., protocol, recruitment materials, consent form, etc.) to the IRB for review and approval prior to initiation of the change(s), except where necessary to eliminate apparent immediate hazards to the subject(s). Changes made to eliminate apparent immediate hazards to subjects must be reported to the IRB within 24 hours.
2. Submission in writing of any and all adverse event(s) that occur during the course of this project that are both serious and unexpected within 10 working/14 calendar days of notification of event.
3. Submission in writing of any and all unanticipated problems involving risks to subjects or others.
4. Use of only IRB approved copies of the consent form(s), questionnaire(s), letter(s), advertisement(s), etc. in your research. Do not use expired consent forms.
5. Informing all physicians listed on the project of changes, adverse events, and unanticipated problems.

The IRB can and will terminate projects that are not in compliance with these requirements. Direct questions, correspondence and forms (e.g., continuing reviews, amendments, adverse events, safety reports) to Ruth Benjamin, (617) 525-3191.

cc: Ellen Kornell, Medical Services, CNY 149 5022

MASSACHUSETTS GENERAL HOSPITAL
